# Carbonic anhydrase inhibitors limit complications in X-linked retinoschisis

**DOI:** 10.3389/fmed.2023.1281068

**Published:** 2023-11-06

**Authors:** Stephanie Wey, Daniel A. Brill, Virginia Miraldi Utz, Robert A. Sisk

**Affiliations:** ^1^Department of Ophthalmology, Abrahamson Pediatric Eye Institute, Cincinnati Children’s Hospital Medical Center, Cincinnati, OH, United States; ^2^Cincinnati Eye Institute, Cincinnati, OH, United States; ^3^Department of Ophthalmology, University of Cincinnati, Cincinnati, OH, United States

**Keywords:** acetazolamide, carbonic anhydrase inhibitors, dorzolamide, juvenile X-linked retinoschisis, X-linked retinoschisis

## Abstract

**Purpose:**

Carbonic anhydrase inhibitors (CAIs) reduce macular schisis in patients with X-linked retinoschisis (XLRS). The purpose of this study was to determine if CAIs reduce the incidence of complications from XLRS, including macular atrophy, retinal tears, and retinal detachment (RD), the most common causes of vision loss in patients with XLRS.

**Methods:**

For this retrospective interventional case series, a chart review of patients examined at Cincinnati Children’s Hospital Medical Center [CCHMC] and Cincinnati Eye Institute [CEI] between 1/1/2015 and 1/16/2023 was performed. Male patients were included based on genetically-confirmed *RS1* or typical clinical presentation with known family history of XLRS with at least two follow-up visits.

**Results:**

Twenty-eight patients (56 eyes) with XLRS were included. There were 10 *RS1* variants among the 21 genotyped patients. Median age at clinical diagnosis was 10.4 years old (range: 0.4–55.7 years) with median follow-up time of 4.7 years (range: 0.2–38.3 years). Median presenting Snellen visual acuity was 20/60 (logMAR 0.48, range: 0.18–3). In 26 eyes of 15 patients treated with CAIs, median CST pre-treatment was 416 microns (range: 198–701 microns), and median percentage decrease in CST on treatment was 21.8% (range: 0–74.5%) from highest pre-treatment CST. Reduction in CST with CAI use was statistically significant (*p* = 0.02), but not logMAR VA (*p* = 0.64). There was no significant difference in CST between patients treated with topical vs. oral CAI (*p* = 0.95) or between patients with partial or complete CAI adherence (*p* = 0.60). Ten eyes of seven patients had an RD requiring surgical intervention. No treated eyes developed new macular atrophy, peripheral retinoschisis, retinal tears, or RD; two eyes on topical CAIs had spontaneous resolution of bullous peripheral retinoschisis.

**Conclusion:**

During the follow-up period, patients taking CAIs reduced macular schisis and did not experience new complications of macular atrophy, retinal tears, or RD. This is a relatively large cohort with long-term follow-up periods for patients with XLRS. Reduced macular schisis may not require perfect adherence with CAIs. A large, prospective, randomized, controlled clinical trial is needed to determine the potential of CAIs to improve visual function, reduce retinoschisis, and prevent RD.

## Introduction

X-linked retinoschisis (XLRS), also known as congenital or juvenile XLRS, is an inherited retinal disease found in approximately 1:10,000 males (1) caused by variants in retinochisin (*RS1*) (2). *RS1* plays an important function in retinal bipolar and photoreceptor cell adhesion and signal transduction (3). Macular retinoschisis, observed in 99% of XLRS patients, reduces visual acuity in childhood until cystic cavities collapse and macular atrophy develops at a mean age of 25 years (4). Recent swept-source optical coherence tomography angiography demonstrates pathologic loss of flow in the deep capillary plexus accompany advancing macular schisis and atrophy (5). Peripheral retinoschisis is present in 50% of XLRS patients (5), favors the inferotemporal quadrant, and is associated with rhegmatogenous retinal detachment (RD) in 10–22% of patients (6).

Currently, limited treatment options are available to prevent macular atrophy and RD in XLRS patients. While gene therapy appears promising for other inherited retinal diseases, initial Phase I/II intravitreal gene therapy clinical trials (NCT02416622) failed to reduce retinoschisis or demonstrate improved outcomes compared to the natural history of disease (7). Several other gene-based trials for XLRS are ongoing (NCT05878860, NCT02317887, NCT05814952). In several small case series, both oral acetazolamide and topical dorzolamide have been found to reduce foveal schisis and improve vision (8–21). The purpose of this study is to investigate whether carbonic anhydrase inhibitors (CAIs) reduce the incidence of complications from XLRS, including macular atrophy, retinal tears, and retinal detachment (RD).

## Methods

### Study population

This was a retrospective case series approved by the institutional review board at the Cincinnati Children’s Hospital Medical Center and conducted in adherence with the Health Insurance Portability and Accountability Act of 1996 and the tenets of the Declaration of Helsinki. A query of ICD-10 diagnosis code H33.103 was performed on medical records of all patients from Cincinnati Eye Institute and Cincinnati Children’s Hospital Medical Center who had an office visit between 1/1/2015 and 1/16/2023. Patients were included based on the following: genetically-confirmed *RS1* or typical clinical presentation with known family history of XLRS with at least two follow-up visits since initial diagnosis. All XLRS patients with macular schisis and without macular atrophy were treated with topical or oral CAIs. Generally, topical CAIs were the preferred initial management to avoid systemic side effects common to acetazolamide. Oral acetazolamide was used first when parents refused to administer topical CAIs.

### Data collection

The medical records of each patient were reviewed, the earliest dated 9/28/1994. Data collected included age at clinical diagnosis, genetic testing results including mutation and protein, mean central subfield thickness (CST) measured on spectral domain optical coherence tomography (OCT, Heidelberg Spectralis^®^ HRA-OCT), CAI treatment type (oral acetazolamide, dorzolamide, brinzolamide, or combination), CAI dose and frequency, CAI side effects, surgical history, visual acuity (VA), presence of macular schisis, macular atrophy, peripheral retinoschisis, retinal tear, and retinal detachment based on clinical exam as well as OCT and fundus autofluorescence. If the self-reported treatment did not correspond to the prescribed frequency as documented in the previous chart note or patient or family admitted to missing dosages, this was considered incomplete adherence to treatment.

### Data analysis

All statistical analyses were performed on Microsoft Excel (version 16.16.22, Microsoft Inc., Redmond, WA). Patients with incomplete data were excluded from the specific sub-analyses. Given XLRS is a bilateral process, inter-eye correlation was calculated using intra-class coefficient (22). For the purposes of data analysis, VA was converted to logMAR. When VA was documented as counting fingers, hand motion, light perception, or no light perception, it was converted based on a previously reported scale (23). *T*-test was used for comparing continuous variables, and chi-squared test was used for comparing categorical variables. A *p*-value of < 0.05 was considered statistically significant, with Bonferroni correction applied when more than two hypothetical tests were analyzed. Continuous data were represented as means with standard deviations and median with ranges. Categorical data were expressed as proportions.

## Results

### Patient demographics

Twenty-eight patients with XLRS met the inclusion criteria (Table 1). All patients were male and Caucasian. The median age at the time of clinical diagnosis was 10.4 years old (range: 0.4–55.7 years). The median presenting logMAR VA was 0.48 (Snellen acuity 20/60, range 0.18–3) for 52 eyes of 26 patients; two pre-verbal patients were tested with CSM. Median follow-up time since diagnosis was 4.7 years (range: 0.2–38.4 years) for 27 patients; one patient had inconsistent documentation prior to electronic medical records. Excluding 4 patients with macular atrophy, 24 patients were treated with CAIs, including 37 eyes of 19 patients treated with topical CAIs and 14 eyes of 7 patients treated with acetazolamide. Median follow-up time from the initiation of treatment was 2.9 years (range: 0–17 years) for 23 patients-one patient’s start date was unknown. Intra-class coefficient of VA and pre-treatment CST was 0.31 and 0.36, respectively. As such, each eye was treated independently for analysis.

**Table 1:** Clinical characteristics of our study cohort.

**Age at diagnosis (years, *n* = 28 patients)**	
Mean (SD)	15.7 (15.6)
Median (range)	10.4 (0.4, 55.7)
**Follow-up from clinical diagnosis (years, *n* = 27 patients)**	
Mean (SD)	9.4 (9.7)
Median (range)	4.7 (0.2, 38.4)
**Visual acuity on presentation (logMAR, *n* = 52 eyes)**	
Mean (SD)	0.59 (0.49)
Median (range)	0.48 (0.18)
Intra-class coefficient	0.31
Presence of peripheral bullous schisis (percentage, *n* = 56 eyes)	37.5%
**CAI use (number and percentage, *n* = 56 eyes)**	
Systemic	14 (25.0%)
Topical	37 (66.1%)
Concurrent	1 (1.8%)
Sequential	2 (3.6%)
**Follow-up from initiation of treatment (years, *n* = 23 patients)**	
Mean (SD)	4.3 (4.6)
Median (range)	2.9 (0, 17)

### Molecular characteristics

Ten *RS1* disease-causing variants were identified (*n* = 21 patients) (Table 2). The most prevalent pathogenic variant was c.608 C > T, p.(P203L) (*n* = 5 patients). With Bonferroni-corrected alpha level of 0.002, there was no significant difference in rates of retinal tear (*p* = 0.02), peripheral retinoschisis (*p* = 0.83) or RD (*p* = 0.27) among the variants with respect to genotype. Twenty-one of 29 patients had pathogenic *RS1* variants identified by genetic testing. All remaining patients met the definition for clinical diagnosis and had consistent features with an X-linked pedigree with known affected family members.

**Table 2:** Genotype–phenotype characteristics of patients with positive genetic testing.

No. of patients	Genotype	Protein	ACMG variant classification	(+) Retinal tear	(+) Peripheral retinoschisis	(+) Retinal detachment
2	c.305G > A	p.Q102R	Pathogenic	1 patient (2 eyes)	1 patient (2 eyes)	1 patient (2 eyes)
1	c.329G > A	p.C110Y	Pathogenic	0	1 patient (1 eye)	0
4 (2 families)	c.325G > A	p.G109R	Pathogenic	0	0	0
3 (2 families)	c.590G > A	p.R197H	Pathogenic	2 patients (3 eyes)	2 patients (3 eyes)	2 patients (3 eyes)
2	c.579dup	p.I194H	Pathogenic	0	1 patient (2 eyes)	1 patient (2 eyes)
5 (3 families)	c.608C > T	p.P203L	Pathogenic	0	3 patients (5 eyes)	0
1	c.626G > A	p.R209H	Pathogenic	0	0	0
1	c.574C > T	p.P192S	Pathogenic	0	0	1 patient (1 eye)
1	Large hemizygous deletion encompassing exons 2 and 3	–	Unknown	0	1 patient (1 eye)	1 patient (1 eye)
1	c.(52 + 1_53-1)_(78 + 1_79-1)del	–	Unknown	0	0	0

### Treatment with carbonic anhydrase inhibitors

Twenty-six eyes of 15 patients with macular schisis without macular atrophy treated with CAIs were analyzed (Table 3). Twenty eyes (77%) were treated with a topical CAI, usually with dorzolamide 2% two to three times daily. One patient was treated with brinzolamide 1% three times daily due to a temporary dorzolamide shortage for 6 months. Four of the 15 patients were treated with oral acetazolamide 250 mg twice daily. The median CST prior to treatment was 416 microns (range: 198–701 microns). The median reduction in CST while on CAIs was 21.8% (range: 0–74.5%) compared to highest pre-treatment CST. The median lowest CST on treatment was 332 microns (range: 179–427 microns). There was a significant reduction in CST with CAI treatment (*p* = 0.03). There was no significant change in logMAR VA with treatment (*p* = 0.64). There was no significant difference in CST between patients treated with topical vs. oral CAI (*p* = 0.95).

**Table 3:** Clinical outcomes in patients treated with CAIs.

**CST prior to treatment initiation (microns)**	
**Overall (*n* = 26 eyes)**	
Mean (SD)	404 (116)
Median (range)	416 (198, 701)
Intraclass coefficient	0.36
**Adherent (*n* = 17 eyes)**	
Mean (SD)	384 (130)
Median (range)	348 (198, 701)
**Highest CST prior to treatment initiation (microns)**	
**Overall (*n* = 26 eyes)**	
Mean (SD)	433 (105)
Median (range)	438 (227, 701)
**Adherent (*n* = 17 eyes)**	
Mean (SD)	420 (121)
Median (range)	387 (227, 701)
**Lowest CST on CAI (microns)**	
**Overall (*n* = 26 eyes)**	
Mean (SD)	320 (63)
Median (range)	332 (179, 427)
**Adherent (*n* = 17 eyes)**	
Mean (SD)	309 (67)
Median (range)	329 (179, 419)
**CST Reduction with CAI use (microns)**	
**Overall (*n* = 26 eyes)**	
Mean (SD)	−84.0 (106.3)
Median (range)	−63 (522, 0)
**Adherent (*n* = 17 eyes)**	
Mean (SD)	−74.5 (128.4)
Median (range)	−17 (−522, 0)
**Percent change in CST with CAI use**	
**Overall (*n* = 26 eyes)**	
Mean (SD)	−17.5 (16.0)
Median (range)	−14.6 (−74.4, 0)
**Adherent (*n* = 17 eyes)**	
Mean (SD)	−14.0 (18.8)
Median (range)	−5.2 (−74.4, 0)
**Percent change from highest CST with CAI use**	
**Overall (*n* = 26 eyes)**	
Mean (SD)	23.5 (14.7)
Median (range)	21.8 (0, 74.5)
**Adherent (*n* = 17 eyes)**	
Mean (SD)	22.6 (17.9)
Median (range)	20.6 (0, 74.5)
***p*-values of pre- vs. post-initiation of CAIs (paired *t*-test)**	
Overall	
CST	0.03
BCVA	0.64
Adherent	
CST	0.09
BCVA	0.24
***p*-values of comparison analyses (*t*-test)**	
**Adherent vs. non-adherent**	
Change in CST	0.60
**Topical vs. systemic**	
Change in CST	0.95

### Side effects and tolerance of carbonic anhydrase inhibitors

Three patients (20%) experienced worsening macular schisis despite reported medication adherence. One patient was switched from dorzolamide to acetazolamide, but ultimately remained on topical therapy due to intolerance to acetazolamide. One patient switched successfully from dorzolamide to acetazolamide due to lack of response with topical therapy. Another patient was briefly treated with brinzolamide during a dorzolamide shortage. Acetazolamide was added to topical therapy for the third individual with improvement in CST. The reported side effects to acetazolamide were nausea (*n* = 1) and nephrolithiasis (*n* = 1). Stinging sensation was the only reported side effect of dorzolamide (*n* = 3).

### Treatment adherence

Ten patients (17 eyes), or 35% of the study cohort, reported consistent adherence with CAI use based on chart documentation. For those who reported adherence, there was no median change in visual acuity (range: −0.2 to +0.3). The median minimum CST on treatment was 329 microns (range: 179–419 microns), improved from median highest CST of 387 microns (range: 227–701 microns). The median greatest difference in CST before and while on treatment was-17 microns (range: 0 to −522 microns). The median reduction in CST with CAI treatment was −5.2% (range: 0% to −74.4%). There was no statistically significant difference in the maximum change in CST on treatment between complete and incomplete adherence groups (*p* = 0.60). There was no statistically significant difference in the length of follow-up between adherent and non-adherent patients (*p* = 0.24). Anecdotally, dorzolamide adherence was improved with associated reductions in macular schisis by refrigerating the medication and/or applying ice packs over the eyes prior to application.

### Retinal detachment and retinal tears

All patients with an retinal detachment (RD) presented at their first clinic visit with an RD. Eleven eyes of eight patients had a retinal tear (RT) or RD. For this analysis, 10 eyes of seven patients were included; one eye from one patient was excluded as the RD was thought to be due to retinal holes in underlying lattice degeneration rather than within RS or associated with RT (Table 4). Presenting median VA was 20/150 (logMAR 0.9, range: 0.30–3), and final median VA was approximately 20/400 (logMAR 1.25, range: 0.40–3). Excluding one individual whose date of retinal detachment was unknown, median age for onset of RD was 9 years (range: 0.4–20.9 years). No patients developed a new RD or RT while using CAIs.

**Table 4:** Clinical characteristics of patients with retinal complications.

**Age at time of retinal detachment (years, *n* = 10 eyes)**	
Mean (SD)	10.4 (9.0)
Median (range)	9 (0.4, 20.9)
**Etiology (*n* = 10 eyes)**	
Combined	3 (30%)
Rhegmatogenous	3 (30%)
Schisis	2 (20%)
Tractional	1 (10%)
**Visual acuity (logMAR, *n* = 10 eyes)**	
**Initial presentation**	
Mean (SD)	1.18 (0.93)
Median (range)	1.25 (0.40, 3)
**Final presentation**	
Mean (SD)	1.53 (1.41)
Median (range)	0.9 (0.3, 3)
***p*-values of comparison analyses among genetic mutations (chi-squared test)**	
Retinal tear	0.02
Retinal detachment	0.27
Bullous schisis	0.83

### Peripheral retinoschisis

Nineteen eyes of 12 patients (37.5% of eyes in the study cohort) had peripheral retinoschisis. No eyes developed new peripheral retinoschisis while receiving treatment with CAIs. Notably, peripheral retinoschisis resolved in two eyes of two patients while on treatment with CAIs at 0.8 and 2.3 years, respectively.

## Discussion

Without intervention, vision loss is inevitable in patients with XLRS from macular atrophy or retinal detachment, although the severity and timing of vision loss varies among patients (4). No patient developed these complications while using CAIs, which is notable considering the relatively large cohort size, long follow-up period, and variable patient adherence during the study period. Our series confirmed findings from other studies that CAIs reduce macular schisis (8–21) and possibly peripheral schisis (17). Some case studies report improved VA while on CAIs (9, 11, 12, 18, 21), which suggests reducing macular schisis preserves macular function. No statistical improvement in VA for our population was observed, which may be related to the high rate of reduced compliance, dysfunction from inadequate retinoschisin, incomplete resolution of macular schisis, or continued progression of macular degeneration from XLRS (4). We suspect that consistent, long-term suppression of macular schisis may yield better visual outcomes or delay macular atrophy, although this could not be concluded from our study. However, as there are likely multiple mechanisms underlying retinoschisis, such as loss of structural integrity, pump failure, and traction (24), CAIs only address some of these mechanisms, so complete resolution of retinoschisis may not be possible depending upon the chronicity of disease and the degree of retinal degeneration or tractional components.

**Figure 1:**
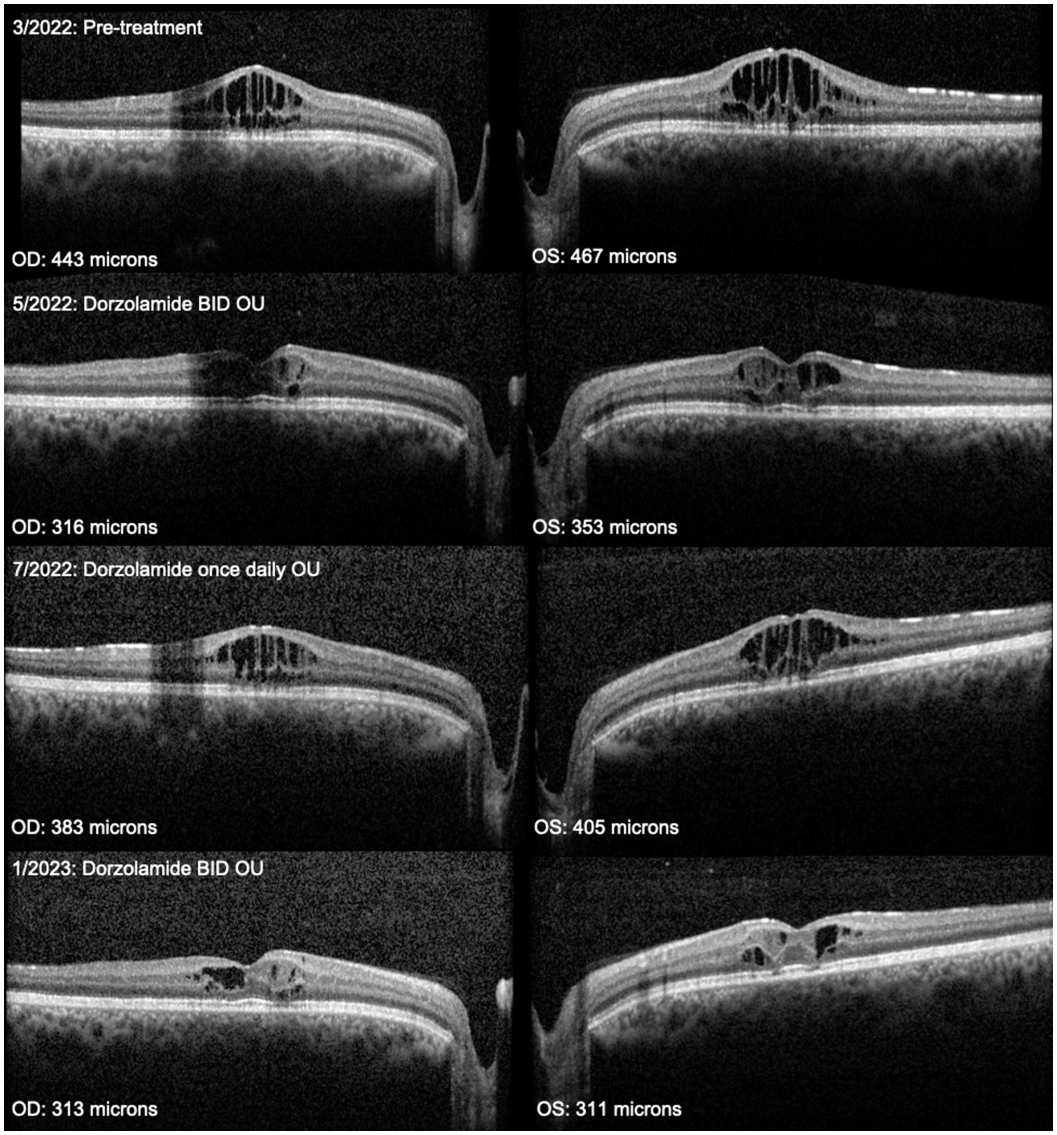
Series of OCT horizontal raster scans through the foveas of the right and left eyes, respectively, of a 22-year-old patient with macular schisis from X-linked retinoschisis treated with topical dorzolamide 2.0%. The central subfield thickness (CST) is listed in the bottom left corner of each panel. Severe foveal schisis initially improved with dorzolamide BID but worsened when reduced adherence averaged once-a-day application between 5/2022 and 7/2022. With several months of improved adherence, foveal schisis continued to improve with reduction in CST.

Adherence with CAI use can be particularly challenging, as side effects are common whether administered topically or orally. Topical dorzolamide is acidic and causes ocular stinging, loss of medication from reflexive tearing, and keratopathy with chronic application. Our series may underreport the incidence of this side effect by its retrospective nature, and we routinely recommended refrigerating dorzolamide, applying artificial tears, and ice packs prior to application to reduce this complaint. Brinzolamide produces less stinging but is more expensive. Oral acetazolamide can cause a myriad of unpleasant symptoms, including numbness and tingling of extremities, abdominal pain, reduced appetite (which is problematic in pediatric patients), and metallic taste to carbonated beverages. More serious concerns of photosensitivity, bone marrow suppression, metabolic acidosis, and electrolyte imbalance require periodic monitoring. In our experience, topical and oral CAIs performed similarly in reducing CST, but risks of oral CAIs greatly outweigh topical. Anecdotally, combining oral and topical CAIs reduced macular schisis when topical or oral CAIs individually were ineffective for our patients with XLRS or retinitis pigmentosa. Even short-term reductions in adherence limited the effectiveness of CAIs in reducing CST (Figure 1). However, the lack of difference between maximum reduction in CST despite differences in adherence support prior literature demonstrating recovery of CAI effect in schisis reduction when adherence is improved (25). We found that patients who reported instilling topical CAIs with complete adherence twice a day had a therapeutic effect. However, because nonadherence was common and most commonly involved missing doses rather than days, we recommend dosing three times a day. In addition to medication intolerance, children represent a unique population as they often depend on parents to administer medications at home. Older children or teens may not be accurately report administering medications; we recommend parental confirmation of CAI adherence. Although we observed short-term visual improvements in some patients on CAIs, the need for frequent, chronic dosing for physiologic effect makes durable improvements in vision and structure challenging. Longer follow-up will be required to determine whether early intervention and improved adherence with CAIs will result in more favorable anatomic and functional outcomes. An unmet therapeutic need is the development of sustained-release CAIs for XLRS and glaucoma management.

Retinal detachment complicating XLRS is surgically challenging to repair, frequently complicated by proliferative vitreoretinopathy, and has a poor prognosis for visual recovery. Therefore, prevention of RD is an important goal in the care of XLRS patients. Reducing peripheral schisis may reduce RD risk beyond trauma avoidance, as a significant component of retinoschisis is vitreous traction, which relates to risk for retinal tears and rhegmatogenous RD (26). Resolution of peripheral schisis in XLRS may occur with posterior vitreous separation spontaneously or through surgical induction during vitrectomy (24). It is noteworthy and encouraging that two patients had resolution of their peripheral retinoschisis while on CAIs, although a causal relationship cannot be established. Furthermore, no patient on CAIs developed a RD, retinal tear, or macular atrophy. In addition to serial macular OCTs, peripheral OCT technology may be helpful to objectively monitor clinical response to CAIs in XLRS patients. Rather than simply determining the presence or absence of peripheral retinoschisis, serial measurements may provide further insight into the peripheral retina’s response to CAIs and the degree of vitreous separation from the peripheral retina.

As with other retrospective case series involving rare diseases, there are inherent limitations of this study. Our intent-to-treat bias excluded a control group. Sample size and limited follow-up limits our conclusions about the impact of CAIs on foveal schisis, visual acuity, and RD prevention. Peripheral fundus imaging and ultrasonography were not performed routinely at each visit. This study is subject to recall bias and relied exclusively on medical records for data collection.

In summary, our series showed CAIs reduce macular schisis in XLRS eyes without a significant change in visual acuity. No patient developed macular atrophy, retinal tear, or RD while taking CAIs, even with reduced adherence, but sample size and duration of follow-up limit conclusions about a preventative effect. A larger multicenter retrospective trial or a prospective, randomized, controlled trial is warranted to explore the efficacy of CAIs in preventing RDs or macular atrophy, reducing macular schisis, and preserving visual acuity in patients with XLRS. Strategies to improve tolerability and adherence to chronic topical CAI therapy are needed.

## Data availability statement

The original contributions presented in the study are included in the article/supplementary material, further inquiries can be directed to the corresponding author.

## Ethics statement

The studies involving humans were approved by Cincinnati Children’s Hospital Institutional Review Board. The studies were conducted in accordance with the local legislation and institutional requirements. Written informed consent for participation was not required from the participants or the participants’ legal guardians/next of kin in accordance with the national legislation and institutional requirements.

## Author contributions

SW: Conceptualization, Data curation, Formal analysis, Investigation, Methodology, Writing – original draft, Writing – review & editing, Visualization. DB: Conceptualization, Data curation, Formal analysis, Investigation, Supervision, Writing – original draft, Writing – review & editing, Project administration. VMU: Data curation, Investigation, Writing – original draft, Writing – review & editing, Visualization. RS: Data curation, Investigation, Writing – original draft, Writing – review & editing, Conceptualization, Formal analysis, Methodology, Supervision, Project administration, Validation.
